# Intervention for improving treatment retention and alcohol-related outcomes in patients with alcohol-related liver disease: a randomised controlled trial

**DOI:** 10.1192/j.eurpsy.2025.432

**Published:** 2025-08-26

**Authors:** E. Caballeria, D. Folch-Sanchez, M. T. Pons-Cabrera, P. Guzmán, N. Cabrera, Ó. García-Pañella, J. Gratacós-Ginès, E. Pose, P. Zuluaga, M. Balcells-Oliveró, R. Bataller, A. Hernández-Rubio, B. Serra, C. Oliveras, H. López-Pelayo

**Affiliations:** 1 Health and Addictions Research Group, Institut d’Investigacions Biomèdiques August Pi i Sunyer (IDIBAPS); 2 Universitat de Barcelona; 3 RIAPad. Red de investigación de atención primaria en adicciones, Instituto de Salut Carlos III; 4 Addictions Unit, Psychiatry Department, Hospital Clínic de Barcelona; 5 CookieBox; 6 Escuela de Nuevas Tecnologías Interactivas, Universitat de Barcelona; 7 Liver Unit, Hospital Clínic de Barcelona; 8 Grupo de Enfermedades hepáticas crónicas: mecanismos moleculares y consecuencias clínicas, Institut d’Investigacions Biomèdiques August Pi i Sunyer (IDIBAPS); 9 Enfermedades Hepáticas y Digestivas, Centro de Investigación Biomédica en Red, Barcelona; 10 Internal Medicine, Hospital Universitari Germans Trias I Pujol, Badalona, Spain; 11 King´s College London, London, United Kingdom; 12 Psychiatry Department, Hospital Clínic de Barcelona, Barcelona; 13 RIAPad. Red de investigación de atención primaria en adicciones, Instituto de Salut Carlos III, Madrid, Spain

## Abstract

**Introduction:**

Quitting alcohol use has been described as the main factor capable of modifying the prognosis of alcohol-related liver disease (ArLD). However, retention to the addiction treatment in these patients is low, and relapse in alcohol use is common. Misconceptions in the patients knowledge of the disease and the treatment impact retention. To improve retention, we have designed a blended intervention consisting in a presential brief intervention combined with a gamified webapp (MyWayUp). The intervention provides information regarding the liver disease and treatment, how to improve the prognosis, healthy lifestyles and how to achieve behavioural changes. The intervention was designed using a co-creation approach, and is based in well-stablished psychological principles (cognitive behavioural therapy, CBT; motivational interviewing, MI; psychoeducation; game-based learning).

**Objectives:**

The main objective is to explore the efficacy of the MyWayUp for improving retention to the addiction treatment in patients with ArLD at six months follow-up. As secondary objectives we explore: retention at 1 and three months, adherence to the treatment (attended visits from the total programmed), patterns of alcohol use and quality of life.

**Methods:**

Prospective, randomised controlled trial, 6 months. Patients with ArLD onset would be invited to participate. If signed the informed consent, they would be randomised to the experimental or control condition. The experimental group would receive the brief intervention and given access to the webapp with a unique access code. Patients in the control group received treatment as usual, and after six months, if they had not adhered to the addiction unit, they would be invited to participate as experimental group. Both groups would be programmed the first visit with a psychiatrist, and followed at months 1, 3 and six after inclusion. The study was blinded for professionals and patients, and only one member in the research team would know the allocation group of each patient.

At baseline, sociodemographical variables were collected, as well as clinical data (presence of comorbidities), pattern of alcohol or other substances use (AUDIT; timeline follow back, TLFB), quality of life (EQ-5D-5L) and functionality (FAST). Several measure were taken at months 1, 3 and six: being active in the treatment at the assessment point (retention); adherence; alcohol and other substance use (TLFB) quality of life and functionality.

**Results:**

The final sample consisted of 82 patients, with a mean age of 55.3 (SD = 11.4). 38.2 % were women, and 53% of the participants were allocated to the experimental group.

**Image 1:**

**Figure:**
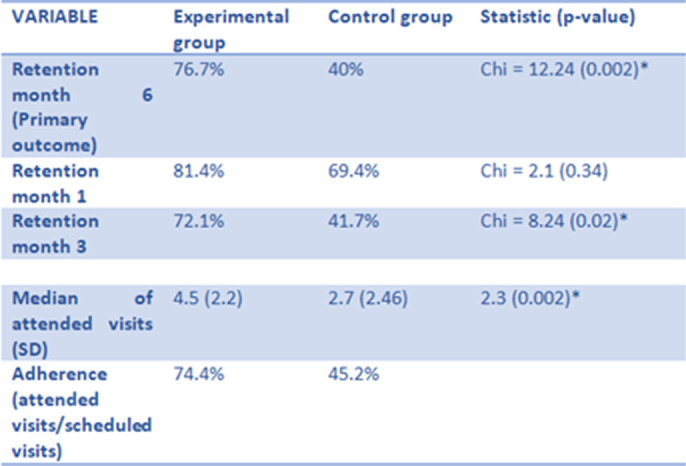

**Image 2:**

**Figure:**
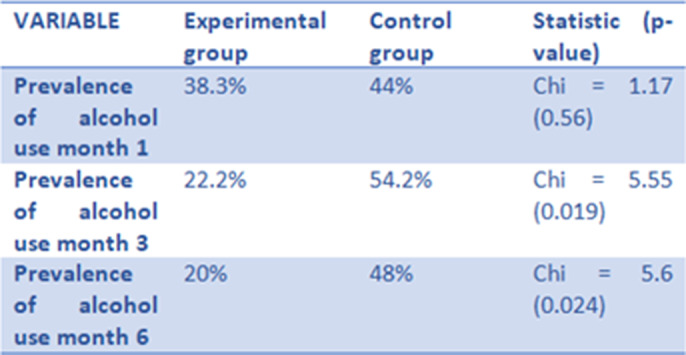

**Conclusions:**

MyWayUp has shown efficay in improving treatment retention and adherence as well as in improving abstinence rates.

**Disclosure of Interest:**

None Declared

